# Brain and Peripheral Atypical Inflammatory Mediators Potentiate Neuroinflammation and Neurodegeneration

**DOI:** 10.3389/fncel.2017.00216

**Published:** 2017-07-24

**Authors:** Duraisamy Kempuraj, Ramasamy Thangavel, Govindhasamy P. Selvakumar, Smita Zaheer, Mohammad E. Ahmed, Sudhanshu P. Raikwar, Haris Zahoor, Daniyal Saeed, Prashant A. Natteru, Shankar Iyer, Asgar Zaheer

**Affiliations:** ^1^Harry S. Truman Memorial Veteran’s Hospital, U.S. Department of Veterans Affairs Columbia, MO, United States; ^2^Department of Neurology and the Center for Translational Neuroscience, School of Medicine, University of Missouri Columbia, MO, United States

**Keywords:** brain, cytokines and chemokines, mast cells, inflammatory mediators, neuroinflammation, neurodegenerative diseases

## Abstract

Neuroinflammatory response is primarily a protective mechanism in the brain. However, excessive and chronic inflammatory responses can lead to deleterious effects involving immune cells, brain cells and signaling molecules. Neuroinflammation induces and accelerates pathogenesis of Parkinson’s disease (PD), Alzheimer’s disease (AD) and Multiple sclerosis (MS). Neuroinflammatory pathways are indicated as novel therapeutic targets for these diseases. Mast cells are immune cells of hematopoietic origin that regulate inflammation and upon activation release many proinflammatory mediators in systemic and central nervous system (CNS) inflammatory conditions. In addition, inflammatory mediators released from activated glial cells induce neurodegeneration in the brain. Systemic inflammation-derived proinflammatory cytokines/chemokines and other factors cause a breach in the blood brain-barrier (BBB) thereby allowing for the entry of immune/inflammatory cells including mast cell progenitors, mast cells and proinflammatory cytokines and chemokines into the brain. These peripheral-derived factors and intrinsically generated cytokines/chemokines, α-synuclein, corticotropin-releasing hormone (CRH), substance P (SP), beta amyloid 1–42 (Aβ1–42) peptide and amyloid precursor proteins can activate glial cells, T-cells and mast cells in the brain can induce additional release of inflammatory and neurotoxic molecules contributing to chronic neuroinflammation and neuronal death. The glia maturation factor (GMF), a proinflammatory protein discovered in our laboratory released from glia, activates mast cells to release inflammatory cytokines and chemokines. Chronic increase in the proinflammatory mediators induces neurotoxic Aβ and plaque formation in AD brains and neurodegeneration in PD brains. Glial cells, mast cells and T-cells can reactivate each other in neuroinflammatory conditions in the brain and augment neuroinflammation. Further, inflammatory mediators from the brain can also enter into the peripheral system through defective BBB, recruit immune cells into the brain, and exacerbate neuroinflammation. We suggest that mast cell-associated inflammatory mediators from systemic inflammation and brain could augment neuroinflammation and neurodegeneration in the brain. This review article addresses the role of some atypical inflammatory mediators that are associated with mast cell inflammation and their activation of glial cells to induce neurodegeneration.

## Introduction

Neuroinflammation is an inherent host-defense mechanism to protect and restore the normal structure and function of the brain against infection and injury (More et al., 2013). In neurodegenerative diseases, neuroinflammation initially clears up infection to control the disease severity and progression. However, neuroinflammation also acts as a double-edged sword (Kielian, 2016). On the one side, neuroinflammation induces and/or aggravates neurodegeneration in the central nervous system (CNS), while on the other side, it favors the recovery of the injured neurons (Lucas et al., 2006). A recent study indicates that neuroinflammation promotes regeneration to some extent (Bollaerts et al., 2017). Chronic inflammation induces cytotoxic effects and increases the severity of neurodegenerative disease symptoms. Neuroinflammation is the leading cause of devastating neurodegenerative diseases such as Parkinson’s disease (PD), Alzheimer’s disease (AD), Amyotrophic lateral sclerosis (ALS) and Multiple sclerosis (MS)/Experimental autoimmune encephalomyelitis (Chen et al., 2016; Kempuraj et al., 2016b). The activated glial cells release several pro-inflammatory cytokines, chemokines and reactive oxygen species (ROS), which mediate neuroinflammation-induced neurodegenerative disease mechanisms (Kempuraj et al., 2016b). Anti-inflammatory cytokines synthesized by regulatory T cells and neuropeptides secreted by neurons and other cells protect the neurons against neuroinflammation, which helps alleviate the symptoms of neurodegenerative diseases. Recent evidence supports that neuroinflammatory processes involving immune cells, glial cells and neuronal cells are very crucial and fundamental to understanding the origin or pathogenesis of a disease, such as a progressive loss of dopaminergic neurons in PD (Chung et al., 2010; More et al., 2013; Kong et al., 2017; Visan, 2017). Additionally, peripheral inflammation also increases brain inflammatory responses through several mechanisms (Machado et al., 2011; Hernández-Romero et al., 2012; Takeda et al., 2013). Previous studies have shown that non-steroidal anti-inflammatory drugs (NSAIDs), statins and other drugs provide some relief although they are ineffective for neurodegenerative diseases (Szekely and Zandi, 2010; Lehrer, 2014; Kure et al., 2017). This is because the NSAIDs do not efficiently cross the blood brain-barrier (BBB) and do not attain therapeutic levels that are necessary to overcome and reverse neuroinflammation. Therefore, therapeutic options like nasal NSAIDs have been suggested to improve its bioavailability and effectiveness in neuroinflammation by increasing the concentration in the brain (Lehrer, 2014).

The existence of bidirectional communication between the brain and the peripheral immune system has been known over the past two decades (Johnson et al., 1997; Dantzer et al., 2000; Holmes and Butchart, 2011; Hernández-Romero et al., 2012; McCusker and Kelley, 2013; Träger and Tabrizi, 2013; Engelhardt et al., 2017). Neuroinflammation is known to directly and systemically contribute to the recruitment of peripheral inflammatory cells and increase BBB permeability in the neurodegenerative diseases (Hayley and Anisman, 2005; Ferrari and Tarelli, 2011; Espinosa-Oliva et al., 2014; Dendrou et al., 2016; McManus and Heneka, 2017; Schain and Kreisl, 2017). Peripheral infection and inflammation increases BBB permeability and neuroinflammation in the animal model of AD (Takeda et al., 2013). Systemic inflammation also affects hippocampus-dependent memory (Culley et al., 2014; Takeda et al., 2014). Microglia, the macrophage of the brain, is an important resident innate immune cell in the brain. Activation of microglia induces their proliferation in neuroinflammatory conditions (Perry et al., 2010; Perry, 2016). Additionally, astrocytes, neurons, brain pericytes, T cells and mast cells also play a significant role in the brain inflammatory mechanisms. Inflammatory mediators, neurotoxic mediators, inflammasomes and hormones released by the activated glial cells and injured neurons are involved in neuroinflammation in AD and PD pathogenesis (Tufekci et al., 2012; Pennisi et al., 2016; Becher et al., 2017; Gualtierotti et al., 2017; Rustenhoven et al., 2017; Sawikr et al., 2017; Song et al., 2017). Substantia nigra has the highest density of microglial cells, which are found, to be activated in PD brains. In AD, activated microglial cells are found surrounding the beta-amyloid (Aβ) plaques. Microglial activation and inhibition of microglial activation is tightly regulated by a number of microRNAs including pro-inflammatory mir-155 and mir-204 as well as anti-inflammatory mir-203, mir-181c and mir-424 (Cardoso et al., 2012; Li L. et al., 2015). More recently, using the 1-methyl-4-phenyl-1,2,3,6-tetrahydropyridine (MPTP) mouse PD model, a study has shown that 1-methyl-4-phenylpyridinium (MPP^+^) downregulates mir-7116-5p in microglia and potentiates TNF-α production and inflammatory responses leading to DA neuron damage (He et al., 2017).

In addition to significant brain innate immune system activation, peripheral innate immune system activation is recognized as a prominent feature contributing to the severity of neuroinflammatory diseases progression (Träger and Tabrizi, 2013). Systemic inflammation exacerbates clinical symptoms, neuroinflammation and neuronal death in older people and in patients who have suffered stroke and chronic neurodegenerative disease (Teeling and Perry, 2009). Systemic infection and increased IL-1β have been reported to induce a cognitive decline in AD patients (Holmes et al., 2003). Systemic inflammation could induce behavioral and cognitive changes and accelerate neurodegenerative disease pathogenesis (Perry, 2004; Perry et al., 2007; Cunningham et al., 2009). Excess accumulation of activated immune cells in the brain has been well documented in studies covering multiple neurodegenerative diseases. Inflammatory mediator levels in the plasma are elevated in neuroinflammatory conditions. Thus, cytokine levels can be used as a biomarker for neuroinflammatory diseases including PD (Litteljohn and Hayley, 2012). Cytokine and chemokine levels correlate between cerebrospinal fluid (CSF) and plasma in AD (Sun et al., 2003) and PD (Blum-Degen et al., 1995). This indicates that similar type of immune responses occur in the brain and periphery, and show the communication between the brain and peripheral immune systems. An interesting study demonstrated upregulation of nuclear factor-kappaB (NF-κB) activation in the peripheral blood mononuclear cells in AD patients thereby suggesting NF-κB inhibitors as therapeutic agents in AD patients (Ascolani et al., 2012). Imbalance of peripheral T cell subsets (decreased CD8^+^ and increased CD4^+^ cells) has been reported in AD patients indicating peripheral immune response in AD (Shalit et al., 1995); this is similar to a previous report on brain tumor patients that showed altered peripheral immune parameters in patients with a brain tumor (Kempuraj et al., 2004a). Increased TNF-α levels due to either acute or chronic systemic inflammation have been shown to increase cognitive decline in AD (Holmes et al., 2009) and neurodegeneration in PD (Litteljohn et al., 2010). A previous study has shown that systemic injection of TNF-α into an adult mouse activates brain microglia to produce proinflammatory mediators that induces death of dopaminergic neurons in the substantia nigra (Qin et al., 2007), and this demonstrates a direct link between systemic inflammatory factors and neurodegeneration in the brain. Moreover, during sustained immune response in the brain, several multifunctional pro inflammatory factors, mast cells and T cells from the periphery migrate to the brain, and go directly to or through glial cells while neurons augment and maintain chronic neuroinflammatory responses leading to neurodegeneration (Prinz and Priller, 2017). However, the communication between peripheral inflammatory factors, brain inflammatory factors and the cells in neuroinflammation is not yet clearly understood. Thus, no effective treatment options are currently available for neurodegenerative diseases. Multiple studies have demonstrated that systemic inflammation induces onset and progression of neuroinflammation and neurodegeneration in the CNS. Mast cells play an important role in the systemic inflammation by releasing various inflammatory, neuroactive and vasoactive mediators. Several previous review articles have shown how inflammatory mediators such as IL-1, TNF-α, IL-6, chemokine (C-C motif) ligand 2 (CCL2), ROS released by microglia, T-cells and mast cells induce neuroinflammation. Although mast cells release many neuroinflammatory mediators, we will focus only on select atypical mediators in this review article. This review article will focus on the mast cell associated peripheral and brain inflammatory mediators and mast cells that migrate into the brain and either directly or through astrocytes, microglia and neurons induce and sustain neuroinflammation leading to neuronal death.

## Mast Cells in Neuroinflammation

**Figure 1:**
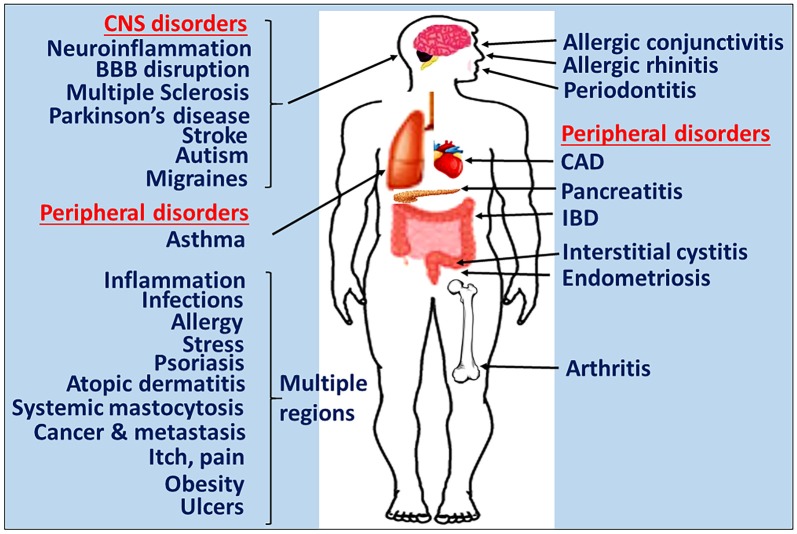
Mast cells in inflammatory diseases. Mast cells play a crucial role in the pathogenesis of many inflammatory diseases of various organs. Certain systemic inflammatory conditions could affect and augment neuroinflammation and neurodegeneration. Therefore, targeting mast cells represents a potentially novel approach to developing the next generation of precision guided therapeutic strategies for the treatment of inflammatory diseases. IBD, inflammatory bowel disease; CAD, coronary artery disease; BBB, blood-brain barrier.

**Figure 2:**
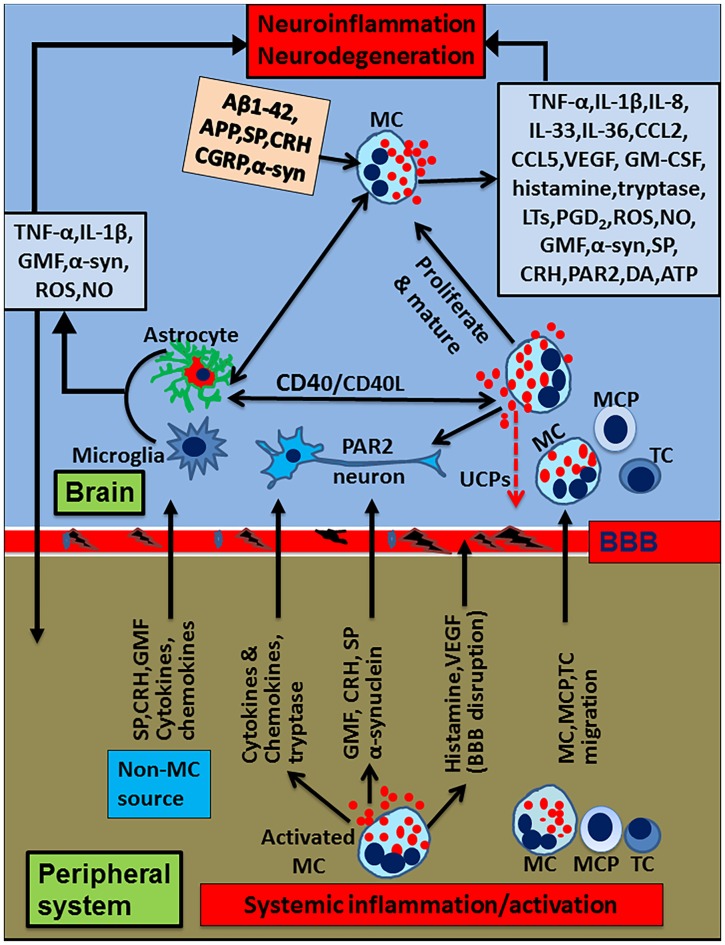
Schematic diagram showing peripheral inflammatory factors and cells on neuroinflammation and neurodegeneration. Peripheral mast cell activation releases proinflammatory and neurotoxic mediators such as histamine, glia maturation factor (GMF), α-synuclein, corticotropin-releasing hormone (CRH), proteases, cytokines and chemokines. These mediators can induce neuroinflammation by inducing BBB breakdown, entering into the brain and activating glia and neurons to secrete various additional inflammatory mediators. Peripheral mast cells and T-cells enter into the brain, proliferate and secrete proinflammatory mediators that activate glia and neurons to secrete more inflammatory mediators, reduce uncoupling protein (UCP) expression, and induce neurodegeneration. Further, glia and mast cells reactivate each other in the brain through co-stimulatory molecules CD40/CD154 or inflammatory mediators such as TNF-α, IL-1β or IL-33. Mast cell tryptase acts on the neurons through PAR2. Mast cells can reactivate by their own mediators in an autocrine and paracrine manner to exacerbate inflammatory mechanisms. α-synuclein or MPP^+^ from glia/neuron or extracellular Aβ1–42 can further activate mast cells to release neuroinflammatory mediators in Alzheimer’s disease (AD) or Parkinson’s disease (PD). Additionally several inflammatory mediators from the peripheral system can alter BBB, enter the brain and activate the neuroinflammatory pathways. Inflammatory mediators released from activated microglia and astrocytes can enter into peripheral system through defective BBB; then, they can activate and recruit immune and inflammatory cells towards the inflammatory site in the brain. MC, mast cell; MCP, mast cell progenitor; TC, T-cell; PAR2, protease activated receptor-2.

Mast cells are crucial in allergic and anaphylactic reactions, acquired and innate immunity, tissue damage and repair processes, and pain mechanisms (Schwartz, 2002; Metz et al., 2007; Galli et al., 2008; Gilfillan et al., 2011; Tete et al., 2012; Saito, 2014; Morita et al., 2016). Mast cells develop from hematopoietic progenitor cells in the bone marrow, move to various tissues and mature into different types of mast cells (Gurish and Austen, 2001). Mast cells are implicated in several allergic and inflammatory diseases including neurodegenerative and demyelinating diseases (Brown and Hatfield, 2012; Conti and Kempuraj, 2016; Russi et al., 2016; Theoharides et al., 2016; Li et al., 2017; Yehya and Torbey, 2017). Mast cells are involved in both peripheral inflammatory conditions and diseases including allergies, asthma, cancer, arthritis, psoriasis, interstitial cystitis, rhinitis, MS, PD, stroke, increased BBB permeability, autism, pain, migraines, sleep disorders, intracerebral hemorrhage, stress and inflammation as shown in Figure 1. The systemic inflammatory conditions and diseases can exacerbate neuroinflammation through several different pathways. Mast cells are multifunctional sensor and effector cells in nervous, vascular and immune system disorders (Ryan et al., 2009; Silver et al., 2010). In the brain, mast cells are primarily located on the brain side of the BBB, and communicate with neurons, glia and neurovascular components by releasing their mediators and through mast cell receptors (Silver and Curley, 2013; Dong et al., 2014). Mast cells are generally seen near the glial cells in neuroinflammatory conditions, indicating their interactions with brain cells in neuroinflammation. Various factors such as species, sex, age and outside environmental conditions controls the number and the extent of its activation in the brain (Silver and Curley, 2013). Additionally, the number of mast cells also vary based upon staining and other detection techniques used. In a physiological condition, the total number of mast cells present in the CNS is limited. But, mast cells are very powerful cells and even a few mast cells can release sufficient quantity of inflammatory mediators that can affect BBB and activate glia and neurons in the CNS (Silver and Curley, 2013). Mast cells release neurotrophic factors such as nerve growth factor (NGF) and facilitate neurotransmission, neuronal growth and survival in the normal brain (Kritas et al., 2014a). However, increased number of mast cell involvement and their activation is deleterious, since this could increase BBB permeability, activate astrocytes, oligodendrocytes, microglia and T cells in neuroinflammation (Zhang et al., 2016). Mast cells are resident in the CNS (Skaper et al., 2014), and also able to cross BBB into the brain from the peripheral tissue in neuroinflammatory conditions (Skaper et al., 2012) as well as in physiological conditions (Silverman et al., 2000). Mast cells can recruit and activate other inflammatory cells and glial cells in the brain at the site of inflammation and induce vasodilation in neuroinflammation as shown in Figure 2 (Nelissen et al., 2013; Yehya and Torbey, 2017).

Mast cells release multifunctional cytokines/chemokines and neuroactive mediators including IL-1β, IL-6, IL-8, IL-18, IL-33, TNF-α, vascular endothelial growth factor (VEGF), corticotropin-releasing hormone (CRH), granulocyte macrophage-colony stimulating factor (GM-CSF), CCL2, dopamine, substance P (SP), histamine, tryptase, prostaglandins (PGD), leukotrienes, ROS, reactive nitrogen species (RNS) and nitric oxide (NO) selectively depending upon the tissue micro environment and the type of stimuli (Mekori and Metcalfe, 2000; Kalesnikoff and Galli, 2008; Sismanopoulos et al., 2012; Theoharides et al., 2012; Kempuraj et al., 2013). Cytokines such as IL-1β and TNF-α in higher concentrations either directly induce neuronal damage or through activating glial cells, and these cytokines are suggested as a drug target in brain injury therapy (Allan and Rothwell, 2001, 2003; Allan et al., 2005; Brough et al., 2015).

We were the first to show that IL-1 can activate mast cells to release inflammatory mediators (Kandere-Grzybowska et al., 2003). Mast cell-derived vasoactive and inflammatory mediators cause vasoconstriction, increased vascular permeability, edema and inflammatory cell recruitment to the site of inflammation. Mast cells are implicated in all stages of inflammation, tissue damage, and tissue repair mechanisms after the inflammatory responses are over in the inflamed tissues (Kim et al., 2011; Skaper et al., 2012, 2013; Zhang et al., 2012). Mast cells are important sensors of cell injury and damage through IL-33 recognition after it is released from the injured cells (Enoksson et al., 2011). Microglia, the resident brain immune cells and mast cells are considered as two important cells that can mediate neuroinflammation in the brain. Moreover, mast cells can respond and release many neuropeptides that are implicated in the process of neuroinflammation (Tore and Tuncel, 2009; Skaper et al., 2012). Mast cells cross-talk with T-cells, astrocytes, oligodendrocytes and microglia during the pathogenesis of chronic neurodegenerative diseases (Skaper and Facci, 2012; Skaper et al., 2012, 2014; Skaper, 2016). Mast cells can communicate with microglia, astrocytes and neurons through CD40L, CD40, toll-like receptor 2 (TLR2), TLR4, protease-activated receptor 2 (PAR2), CXCR4/C-X-C motif chemokine 12 (CXCL12) and C5aR (CD88). Mast cells also influence glia migration and activation associated with inflammatory mediator release (Kim et al., 2011; Skaper et al., 2012, 2013; Zhang et al., 2012).

Mast cells are the major link between neurons and neuroinflammation (Tore and Tuncel, 2009). Furthermore, mast cells and microglia are known to trigger the activation of two different signaling pathways in neuroinflammation (Skaper et al., 2012; Kempuraj et al., 2016b). Mast cells communicate with neurovascular unit components and induce cognitive dysfunction (Li et al., 2017). Our recent report stating that mast cells are activated by the proinflammatory brain protein glia maturation factor (GMF), strengthens the link between mast cells and brain cells in neuroinflammation (Kempuraj et al., 2016a). Astrocytes also express receptors for mast cells histamine in addition to other receptors indicating the interaction between these cells (Hösli et al., 1984). Mast cell degranulation-derived proteases cleave tryptase-activated receptors (PARs) expressed on the neurons in neuroinflammation and neurodegeneration (Saito and Bunnett, 2005). Mast cell granules also contain dopamine that is released upon activation (Freeman et al., 2001). Previous reports demonstrated that mast cell activation induces and promotes the severity of EAE and that mast cell deficient mice show reduced EAE disease severity. Mast cell reconstitutions into mast cell-deficient mice have been used to study the role of mast cells in CNS diseases (Secor et al., 2000; Sayed et al., 2011). Reconstitution of mast cell deficient mice with cultured mouse bone marrow-derived mast cells (BMMCs) generally restores mast cells in the CNS (Johnson et al., 1991). Mast cells could also act outside the CNS to influence its effects inside the CNS as demonstrated in EAE models (Tanzola et al., 2003). Previous study has shown that mast cell reconstitution in mast cell-deficient mice restores conditions similar to wild type mice (Enoksson et al., 2011). Mast cells can communicate with neurons through the cell adhesion molecule-1 (CADM), N-cadherin, and through the transgranulation process (Wilhelm et al., 2005; Héron and Dubayle, 2013). Activated mast cells induce BBB permeability by releasing vasoactive molecules such as histamine, proteases and metalloproteinases (Héron and Dubayle, 2013). Mast cell-derived histamine affects sleep-wakefulness and other basic behavioral functions (Chikahisa et al., 2013). The types and strength of activation signals as well as mast cell phenotype determine the extent of mast cell activation in inflammatory conditions. Mast cell activation leads to acute explosive degranulation (rapid process) with inflammatory mediators released into extracellular spaces, and also by the selective release of cytokines and chemokines (slow process) (Nelissen et al., 2013). Mast cell-derived mediators that could disrupt BBB include CRH, histamine, VEGF, IL-8, TNF-α, tryptase, prostaglandin D2 (PGD_2_), SP, bradykinin, endorphin, endothelin, neurotensin, NO, vasoactive intestinal polypeptide (VIP) and urocortin (Abbott, 2000; Theoharides and Konstantinidou, 2007; Theoharides et al., 2008; Gu et al., 2015).

Aβ1–42 is the major component of amyloid plaques (APs) in an AD brain. Aβ1–42 is known to activate mast cells that could play an important role in AD pathogenesis (Niederhoffer et al., 2009). In fact the presence of Aβ and tau-protein was detected in the mast cells of peripheral organs such as the skin and stomach of patients with AD (Kvetnoi et al., 2003). Dopaminergic neuronal injury may be detected by mast cells since these cells can detect cellular injury through the IL-33 recognition mechanism (Enoksson et al., 2011). We suggest that in addition to the well-known glial cells activation, mast cells activation with subsequent release of proinflammatory cytokines and chemokines from mast cells as well as mast cell-mediated enhanced proinflammatory mediators from the glia could exacerbate neuroinflammatory conditions leading to neurodegeneration in PD and AD. Our recent findings suggest that activation of mast cells by GMF, MPP^+^ and α-synuclein and release of cytokines, chemokines and neurotoxic molecules and the expression of GMF by mast cells indicate new drug targets for PD and other neuroinflammatory conditions (Kempuraj et al., 2015, 2016a). This review article mainly deals with mast cell association with mitochondrial uncoupling proteins (UCPs), α-synuclein, GMF, CRH, SP and dopamine in neuroinflammatory conditions.

## Mitochondrial Uncoupling Protein (UCP) in Neuroinflammation

UCP2 and UCP4, members of the UCP family are neuroprotective by reducing the formation of ROS and are implicated in neurodegeneration and brain injury (Normoyle et al., 2015), AD (Wu et al., 2010) and PD (Andrews et al., 2005; Ho et al., 2012; Peixoto et al., 2013). UCPs reside on the inner mitochondrial membrane where they alter the mitochondrial membrane potential and ROS production and protect neurons (Cardoso et al., 2015). UCP2 and UCP4 regulate intracellular calcium levels and neurotransmission in the neurons. Abnormal expression of UCP2 and UCP4 has been reported in AD (Wu et al., 2009, 2010). Overexpression or activation of UCP2 and UCP4 may be a new strategy for reducing allergic and inflammatory responses since overexpression protects neurons after MPP^+^ and dopamine-induced toxicity (Ho et al., 2012). UCP4 is primarily expressed in the brain (Ramsden et al., 2012). UCP4 is neuroprotective and important in energy homeostasis and is decreased in PD mice model. Overexpression of UCP4 reduced oxidative stress and inhibited apoptosis (Ho et al., 2012; Ramsden et al., 2012). The expression of UCP2 was demonstrated in lymphocytes, dendritic cells, neutrophils and macrophages (Rousset et al., 2006). UCP2^−/−^ mice develop more severe EAE (Aheng et al., 2011). Macrophages from UCP2^−/−^ mice have a greater inflammatory response to lipopolysaccharide (LPS), including increased NF-κB activation and greater cytokine production. Macrophages overexpressing UCP2 produce less ROS in response to LPS (Negre-Salvayre et al., 1997) and have decreased transendothelial migration (Ryu et al., 2004). Both mitochondrial and cytosolic ROS are also produced by mast cells. Inhibition of ROS production reduces mast cell degranulation, indicating that ROS may play a role in regulating exocytosis process.

Recent studies have suggested that UCP2 manipulation could drive microglia towards protective phenotypes (De Simone et al., 2015). UCP2 activity inhibits exocytosis of dopamine-containing vesicles from rat PC12 cells. UCP2 also dampens the inflammatory response of macrophages. We have previously reported the expression of UCP2 by mouse mast cells, and the LAD 2 human mast cell line, and recently showed the expression of UCP4 by mouse mast cells using immunohistochemical staining (Tagen et al., 2009; Kempuraj et al., 2016a). UCP^−/−^ mouse BMMCs have greater release of proinflammatory molecules after both allergic and non-allergic triggers, in addition to having increased histamine content and increased skin vascular permeability (Tagen et al., 2009). Activated BMMCs from UCP2^−/−^ mice have exhibited greater histamine release, whereas overexpression of UCP2 in LAD2 mast cells have reduced histamine release. UCP2^−/−^ BMMCs have also had elevated histamine content and histidine decarboxylase expression (Tagen et al., 2009). UCP2^−/−^ BMMCs have also had greater production of both IL-6 and PGD_2_, as well as extracellular signal-regulated kinase (ERK) phosphorylation, which is known to regulate prostaglandin synthesis (Tagen et al., 2009). UCP2 can regulate mast cell activation in neuroinflammatory conditions. These studies indicate that UCP2 is a novel regulator of mast cell function with a potential for treatment of mast cell-mediated allergic and inflammatory diseases including neurodegenerative and autoimmune diseases (Tagen et al., 2009; Ho et al., 2012). We have recently shown a downregulation of expression of both UCP2 and UCP4 in the parahippocampal gyrus of AD brains (Thangavel et al., 2017). All these reports show that UCPs expression dynamics in mast cells in the peripheral tissue as well as in the CNS could influence the neuroprotective state. Therefore, it would be worthwhile to examine the molecular mechanism/s underlying transcriptional and posttranscriptional regulation of UCP2 and UCP4. Especially, it would be interesting to decipher the potential role of microRNAs in downregulating the expression of UCP2 and UCP4, not only in AD, but also in PD and other neurodegenerative diseases. Furthermore, targeted overexpression of UCP2 and UCP4 in neurodegenerative diseases could potentially establish their therapeutic as well as neuroprotective effects.

## α-Synuclein in Neuroinflammation

α-synuclein is a cytosolic protein expressed in many immune cells and forms Lewy bodies in the PD brain. α-synuclein is the major component in Lewy bodies and it has been identified as a possible biomarker for PD (Si et al., 2017). α-synuclein is produced by cells in the peripheral tissues as well as from the CNS cells; it is also reported in the blood along with CSF (Sui et al., 2014). α-synuclein is present in the neurons and glia of substantia nigra, thalamus, neocortex and hippocampus. α-synuclein is implicated in both physiological and pathological functions. This cytosolic protein plays an important role in the pathogenesis of PD by inducing neuronal death in the substantia nigra. Extracellular α-synuclein can induce neuroinflammatory reactions in glial cells leading to neurodegeneration. Radiolabeled α-synuclein has been demonstrated to move across BBB in both directions and this movement could have important therapeutic significance (Sui et al., 2014). High levels of α-synuclein in blood could contribute to CNS pathology, since the plasma levels of α-synuclein are significantly higher than in the CSF levels (Shi et al., 2014). α-synuclein from neurons enter into the glial cells and induce the secretion of proinflammatory cytokines and chemokines; moreover, this release is directly proportional to the amount of α-synuclein in the glial cells (Lee et al., 2010). Recently, a neuron-to-neuron transfer of α-synuclein aggregates has been reported in the cell culture system as well as in transgenic mice with neuronal progenitor cell grafts (Desplats et al., 2009). The transferred α-synuclein induces Lewy bodies-like inclusion and apoptotic changes in the recipient neurons. Thus, neuronal-derived excess α-synuclein is transferred and accumulates in the neurons and glia, inducing pathological inclusion formation and degenerative changes. An increased level of α-synuclein can directly bind and disrupt mitochondria (Mullin and Schapira, 2013). α-synuclein in other non-CNS immune cells in the peripheral systems could play an important role in the pathogenesis of PD and other synucleinopathies.

The levels of α-synuclein and phagocytosis may be useful biomarkers in PD patients. PD involves α-synuclein pathology and tissue dysfunction in multiple organs. α-synuclein levels were elevated in monocytes and lymphocytes of sporadic PD patients when compared to control subjects. Increased α-synuclein staining was confirmed by immunoblot analysis of a subset of the peripheral blood mononuclear cells. Elevated α-synuclein is not limited to familial PD, but is also observed in sporadic PD. However, reports regarding the expression of α-synuclein in microglial cells and macrophages are conflicting. In an interesting study, Zhou et al. (2016) have utilized the MPTP mouse model of PD to decipher the role of α-synuclein and mir-7 on the activation of NACHT, LRR and PYD domains-containing protein 3 (NLRP3) inflammasome and neuroinflammation. These studies suggest that mir-7 targets NLRP3 expression besides α-synuclein and modulates NLRP3 inflammasome mediated inflammation. Increased α-synuclein expression with age has been reported in macrophages; however, other studies have reported no expression at all. Previous studies have shown that misfolded α-synuclein directly activates microglia inducing the production and release of the proinflammatory cytokines while increasing antioxidant enzyme expression. In the nigrostriatal system, dopamine is a prominent source of ROS. Therefore, the nigrostriatal system may exhibit enhanced vulnerability and be more prone to PD because this region has high levels of oxidative stress and is enriched in α-synuclein as well as dopamine. α-synuclein is the major component of Lewy bodies responsible for Lewy neuritis in sporadic PD, dementia with Lewy Bodies and a Lewy Body variant of AD. Further, α-synuclein is implicated in glia activation, oxidative stress, neuronal dysfunction, neuroinflammation and neurodegeneration (Béraud et al., 2013). We have previously shown that α-synuclein induced chemokine IL-8 release from cultured human mast cells *in vitro* (Kempuraj et al., 2015) indicating that α-synuclein released in the brain could activate mast cells in inflammatory conditions *in vivo*. A peripheral source of α-synuclein could influence neuroinflammation and neurodegeneration in the CNS.

## GMF in Neuroinflammation

GMF is a novel multifunctional, brain-dominant protein first discovered (Lim et al., 1989, 1990a; Kaplan et al., 1991; Zaheer et al., 1993), purified, sequenced, and cloned in our laboratory (Lim et al., 1989, 1990b). GMF is mainly expressed in the glia in the CNS and is an important factor in neuroinflammation and neurodegeneration in the CNS (Zaheer et al., 2007). We have previously reported increased expression of GMF in the CNS of neurodegenerative and autoimmune disorders (Zaheer et al., 2011; Thangavel et al., 2012; Stolmeier et al., 2013; Kempuraj et al., 2016b). More recently we have also shown that upregulation of GMF is associated with downregulation of UCPs in AD brains (Thangavel et al., 2017). Other investigators have previously demonstrated the expression of GMF in the extra CNS cells/tissues such as in macrophages and peripheral organs (Kaimori et al., 2003; Utsuyama et al., 2003). GMF is a proinflammatory mediator released from glial cells (Zaheer et al., 2008a), and GMF-knockout (GMF-KO) mice showed reduced glial activation and significantly suppressed proinflammatory molecules expression after Aβ infusion when compared to wild type mice (Zaheer et al., 2008b). Primary astrocyte and microglia cultures obtained from GMF-KO mice showed a reduced expression of inflammatory cytokines and chemokines compared to glia obtained from wild type cells, and returned to control levels after reconstitution with an adenoviral construct (Zaheer et al., 2008b). GMF activates astrocytes through p38 mitogen-activated protein kinase (MAPK) and NF-κB signaling pathways (Zaheer et al., 2001, 2007). GMF activates both human and mouse mast cells. Further, our previous studies have shown that BMMCs obtained from GMF-KO mice released less CCL2 than BMMCs obtained from wild type mice. We have also shown that lack of GMF in astrocytes increases antioxidant level and reduces the production of ROS in MPP^+^-mediated toxicity (Khan et al., 2014). We have previously shown the expression of GMF in the cultured mouse and human mast cells by immunocytochemistry (Kempuraj et al., 2015). The lack of GMF reduces the release of inflammatory molecules from mast cells. We have shown that GMF, α-synuclein, MPP^+^, and IL-33 significantly increased the release of IL-8 from human mast cells (Kempuraj et al., 2015). Further, we have shown that incubation of human mast cells with IL-33 upregulates the expression of GMF indicating that GMF expression could be increased during mast cell activation in neurodegenerative diseases. Mast cells could release the stored or newly synthesized GMF during the neuroinflammatory conditions along with other proinflammatory molecules in response to MPP^+^, α-synuclein, Aβ or other PD and AD-relevant proinflammatory molecules.

### GMF Expression in Systemic Pathological Conditions

The expression of GMF could be enhanced in several systemic inflammatory diseases. An earlier study has shown that GMF enhances oxidative stress in renal diseases. GMF is normally absent in the kidney but the expression of GMF is inducible in the proximal renal tubules under the stress of proteinuria and in non-brain tissues (Imai et al., 2015). Thymus shows the expression of GMF, which is important in T cell development (Utsuyama et al., 2003). GMF overexpression in non-brain cells increases the vulnerability to oxidative stress and apoptosis (Kaimori et al., 2003). Another study showed up-regulation of GMF expression in the lesions of 246 human serous ovarian carcinoma (SOC) patients and that high GMF expression exhibited lower disease-free and overall survival rates (Li et al., 2010). Other studies have reported that GMF-γ increased in the cardiac ischemia model with inflammation and angiogenesis (Ikeda et al., 2006). Furthermore, GMF-γ is an important regulator of neutrophil chemotaxis (Aerbajinai et al., 2011). GMF released from systemic inflammation could increase the GMF level in the periphery and this increased GMF may enter into the brain and induce or augment neuroinflammation by activating glia, neurons, mast cells and T-cells. Thus, peripheral-derived GMF may also increase the neuroinflammatory mechanisms in neurodegenerative diseases similar to other inflammatory mediators as shown in Figure 2.

## Neuropeptides SP and CRH in Neuroinflammation

SP is a neurotrophic and neuroprotective factor and can directly modulate glial functions in the brain (Severini et al., 2016). SP is an important proinflammatory neuropeptide that can activate glia and induce neurodegeneration in the CNS (Li W. W. et al., 2015). Elevated levels of SP have been reported in the CSF of AD patients when compared with dementias and control subjects. Further, this increase corresponded to the increased level of Aβ1–42 in the CSF of AD patients (Johansson et al., 2015). However, a reduced level of SP was also reported in the brain and CSF of AD patients (Severini et al., 2016). SP concentration in the subsantia nigra is high and this may be responsible for the increased microglia in this region (Wang et al., 2015). Neuropeptides are involved in neurogenic inflammation and many neuropeptides, including SP, induce mast cell activation and release several neuroinflammatory cytokines, chemokines, tryptase and histamine (Nicoletti et al., 2012). SP is the most abundant neuropeptide in the CNS and is distributed in the peripheral tissues. It has been demonstrated that SP enhances human T-cell proliferation responses and causes an increased synthesis of CC and CXC chemokines. In addition, SP and CRH also induce the synthesis and release of cytokines/chemokines such as TNF-α, CCL2, CCL3, CCL5, GM-CSF, CXCL8, proteases tryptase and chymase, histamine, leukotrienes and PGD_2_ from mast cells (Katsanos et al., 2008). SP is a mediator of neuroinflammation as well as a key element in striatonigral circuitry. PD patients and animal models of PD show dopaminergic neuronal death in the substantia nigra region of the brain. SP increases dopaminergic neuronal death directly and is also induced by LPS or MPP^+^ (Wang et al., 2014). Dopamine induces the release of SP in substantia nigra and the SP potentiate dopamine release in a positive feedback pathway mechanism (Thornton et al., 2010). The substantia nigra has bidirectional neuronal connections with the striatum. Striatal neurons innervate the substantia nigra pars compacta, releasing SP and activating inflammatory cells. We and others have previously shown that several neuropeptides such as SP (Cocchiara et al., 1999; Azzolina et al., 2003; Kulka et al., 2008; Taracanova et al., 2017) and CRH (Theoharides et al., 2004; Alysandratos et al., 2012) are known to activate mast cells to release proinflammatory cytokines and chemokines (Kulka et al., 2008; Shaik-Dasthagirisaheb et al., 2013). Mast cell mediators such as histamine in turn activate microglia and release neuroinflammatory mediators in the brain (Zhu et al., 2014). SP and CRH are important mediators of neuroinflammation and BBB dysfunctions (Esposito et al., 2002; Thornton and Vink, 2012). Increased SP levels have been reported in 6-hydroxydopamine hydrochloride (6-OHDA)-induced PD animal models. A recent report shows that an integrated microfluidic immuno chip can be used as a sensitive, rapid and quantitative detection of SP in the serum of neuroinflammatory conditions (Horak et al., 2014). Previous study has shown that treatment with an SP receptor antagonist is neuroprotective in the intra striatal 6-hydroxydopamine-induced PD (Thornton and Vink, 2012). However, some studies reported decreased SP levels in the substantia nigra of PD (Fernandez et al., 1992). SP can activate glial nicotinamide adenine dinucleotide phosphate (NADPH) oxidase to release ROS (Block et al., 2006).

CRH is the principal coordinator of the stress response by activating the hypothalamo-pituitary-adrenal (HPA) axis (Dedic et al., 2017). CRH is released by neurons in the hypothalamus and in extra CNS cells including mast cells (Kempuraj et al., 2004b; Pardon, 2011). CRH could regulate neuroinflammation by increasing the apoptosis of microglia and activation of mast cells (Ock et al., 2006). CRH can activate microglia through CRH R and release inflammatory mediators (Kritas et al., 2014b). Moreover, SP is known to induce the expression of CRH in glia (Hamke et al., 2006). Emotional/chronic stress enhance glial activation and exacerbates neuronal death through inflammation in the substantia nigra of the PD brain (De Pablos et al., 2014). A case report suggested that major stress could have triggered PD in a young woman (Zou et al., 2013). Stress-induced striatal damage, with subsequent worsening of motor symptoms has been reported in animal models of PD. CRH is also implicated in AD pathogenesis including the generation of Aβ (Pardon, 2011; Marcello et al., 2015). CRH could stimulate mast cells and glial activation and their proliferations in neuroinflammation (Theoharides et al., 2013). We have shown the expression of CRH as well as CRH R in mast cells (Kempuraj et al., 2004b; Cao et al., 2005; Papadopoulou et al., 2005). CRH released from activated mast cells could also activate glial cells in neurodegenerative conditions (Kempuraj et al., 2004b). SP also induces CRH receptor expression on mast cells (Asadi et al., 2012). In fact, administration of inflammatory cytokines induces a depressive disorder similar to the one induced by stressors (Anisman et al., 2002; Gibb et al., 2009; Anisman and Hayley, 2012; Hayley et al., 2013). Thus, stressful conditions can activate peripheral mast cells; activate CRH and SP pathways leading to increased BBB permeability, increased glial activation and more severe neuroinflammation and neurodegenerative diseases.

## Dopamine in Neuroinflammation

Neurotransmitter dopamine is implicated in the pathogenesis of PD and in AD (Bisaglia et al., 2014; Nobili et al., 2017). Self-oxidation of dopamine to dopamine quinone, aminochrome and 5,6-indolequinone mediate PD pathogenesis since aminochrome induces mitochondrial dysfunctions (Segura-Aguilar et al., 2014). The dying neurons in PD release neuromelanin, which induces glial cells activation, and augments neuroinflammatory reactions. Substantia nigra dopamine levels are implicated in 6-OHDA induced recruitment of peripheral inflammatory cells into the nigrostriatal regions in the brain (Espinosa-Oliva et al., 2014). Dopamine can activate microglia to release inflammatory mediators and increase oxidative stress as they express receptors for dopamine (Labandeira-Garcia et al., 2013; Lee, 2013). Mast cells also can synthesize and store dopamine in secretory granules and tyrosine hydroxylase, a key precursor enzyme in dopamine biosynthesis (Freeman et al., 2001; Ronnberg et al., 2012). The amount of dopamine synthesis and storage increases along with mast cell maturation (Ronnberg et al., 2012). Mast cells express D1-like dopamine receptors (Mori et al., 2013) and vesicular monoamine transporters (VMAT2). The D1-like dopamine receptor and VMAT2 transport dopamine/MPP^+^ in mast cells could induce the generation of ROS and the release of inflammatory mediators. D1-like receptors play an important role in allergic reactions in the skin through the T-helper (Th2) formation and mast cell activation (Mori et al., 2013). The expression of tyrosine hydroxylase is induced during mast cell maturation (Ronnberg et al., 2012). Mast cell activation can release dopamine along with other inflammatory mediators (Freeman et al., 2001) and augment neuroinflammation. Dopamine influences mast cell interaction with T cells. Thus, peripheral as well as brain mast cell activation can influence neuroinflammatory mechanisms.

## Mechanisms by Which Peripheral Inflammatory Factors and Inflammatory Cells Augment Neuroinflammation

AD patients show abnormalities in peripheral tissues, immune and inflammatory cells in addition to the pathological changes observed in the brain regions (Khan and Alkon, 2014). Drugs targeting neuroinflammation represent next generation of therapeutic agents for treating AD and PD (Bronzuoli et al., 2016; McKenzie et al., 2017). Studies have shown that peripheral inflammation exacerbates neuroinflammation and neurodegeneration in the brain including its substantia nigra region (Perry, 2010; Villarán et al., 2010; Machado et al., 2011; Hernández-Romero et al., 2012; Träger and Tabrizi, 2013). Animal studies have shown systemic inflammation activates microglia in the brain and releases inflammatory mediators (Hoogland et al., 2015). High levels of IL-6, TNF-α and CCL2 in the plasma and serum of AD, PD and MS patients as well as in various systemic inflammatory diseases can induce the neurodegenerative pathways in these diseases (Träger and Tabrizi, 2013). Systemic inflammation also increases BBB permeability in AD (Takeda et al., 2013). Normally BBB, formed by endothelial cells and astrocyte end-feet, restricts transfer of larger molecules and cells into the brain. Cytokines/chemokines and other proinflammatory molecules have been shown to cross BBB by an active transport mechanism (Banks et al., 1995) or through circumventricular organs that lack BBB (Perry et al., 2007). The peripheral immune and inflammatory mediators can interact with brain BBB endothelial cells and induce the release of additional inflammatory molecules including PGD_2_ into the brain (Ek et al., 2001). Systemic immune cells such as T cells can infiltrate into the brain through the BBB via choroid plexus or CSF that could induce neurodegeneration (Ransohoff et al., 2003; Park et al., 2016). Peripheral inflammation is also translated to the brain through the vagus nerve by neural reflex (Tracey, 2009). As the BBB is disrupted in neurodegenerative diseases, the flow of immune cells and inflammatory molecules across the BBB is increased and thereby increases neuroinflammation (Kortekaas et al., 2005; Takeda et al., 2013). Several previous reports indicate that mast cell activation (Karagkouni et al., 2013; Zhang et al., 2016), as well as peripheral inflammation influences BBB disruption to increase the permeability of inflammatory mediators and immune cell infiltration into the brain thereby spreading peripheral inflammation into the brain (Takeda et al., 2013). Peripheral inflammation due to LPS injection increases neuroinflammation without increasing Aβ level in the AD mouse model, indicating neuroinflammation could increase without increasing the Aβ level in the brain (Takeda et al., 2013). A recent article reported that immunotherapy which induces Aβ clearance alone does not improve cognitive improvement in AD patients and indicates the contribution of systemic inflammation in these patients (Goldeck et al., 2016). Activated microglia and astrocytes release inflammatory mediators, which enter the peripheral system through the defective BBB and recruit immune cells to the brain, thereby exacerbating neuroinflammatory mechanisms (Goldeck et al., 2016).

The brain was originally considered as an immunologically privileged organ but now it is well known that the peripheral immune system and the brain communicate through several pathways (Ransohoff and Brown, 2012). We have previously shown that the presence of a tumor in the brain affects peripheral blood immune parameters (Kempuraj et al., 2004a). Several peripheral inflammatory conditions (Figure 1) could activate mast cells and release proinflammatory and neurotoxic mediators such as GMF, α-synuclein, CRH, neurotensin, proteases, cytokines and chemokines. These mediators can cause up-regulation of the neuroinflammatory mechanisms (Träger and Tabrizi, 2013) by the following mechanisms. These mediators can induce BBB breakdown, enter into the brain and activate glia and neurons to release various neuroinflammatory mediators. Further peripheral mast cells, mast cell progenitor cells and T-cells enter into the brain, proliferate, mature and secrete additional inflammatory mediators. Inflammatory mediators released from these cells activate glia and neurons to secrete additional inflammatory mediators including GMF and neuropeptides reduce UCP expression, and induce neuronal damage and neurodegeneration. Further, glia and mast cells reactivate each other in the brain through co-stimulatory molecules CD40/CD154 or inflammatory mediators such as TNF-α, IL-1β or IL-33. Mast cell tryptase can act on the neurons through PAR2. Mast cells can be reactivated by its own mediators in an autocrine and paracrine manner and exacerbate neuroinflammatory pathways. α-synuclein or MPP^+^ from glia or extracellular Aβ1–42 can activate mast cells to release neuroinflammatory mediators in PD and AD, respectively. Additionally, several inflammatory mediators from peripheral tissue can alter BBB, enter the brain and activate various neuroinflammatory pathways. Furthermore, inflammatory mediators released from activated microglia and astrocytes enter into peripheral system through defective BBB, activate, and recruit immune and inflammatory cells towards the inflammatory site in the brain. However, the influence of peripheral factors on neuroinflammation could vary from individual to individual and time to time in the same individual, which is much like the situation where some people develop allergic reactions in a given place, even though all people living in that area are exposed to the same external environment such as pollen in the spring. Additionally, the severity and the duration of allergic reactions also varies in these people. Another example is the cancer metastasis to the brain. Metastasis of cancer also varies from person to person based on the immune condition of patients. This is because; mast cell numbers and the extent of mast cell activation-mediated disease severity also varies from person to person.

## Future Direction

### A New Approach Is Required to Slow Down the Neuronal Aging Process and Enhance the Neuronal Repair Processes to Treat Neurodegenerative Diseases

Regeneration of the neuronal tissue is limited following any CNS injury. The role of neurite outgrowth inhibitors such as Nogo proteins in inhibiting the neuro regeneration has been reported (Wang et al., 2012). However, in order to achieve efficacious treatment options for the neurodegenerative diseases, it is necessary to explore newer approaches to slowdown the aging process of neurons, replace the dead neurons with the new neurons, and also to repair partially damaged neurons by self-recovery/regenerative mechanisms. Our current knowledge on the interactions between the peripheral immune and inflammatory components with the neuroinflammatory mechanism is still limited (Holmes and Butchart, 2011). Currently most of the laboratories working on the neurodegenerative diseases are dealing primarily with the brain mechanisms but not on the bidirectional communications with the peripheral immune and inflammatory mechanisms. Future research should explore the option for the adult neurons to multiply, grow and make neuronal connections in the adult CNS as seen in the fetal stage. A potentially beneficial approach would be to harness the unlimited potential of patient-specific induced pluripotent stem cells to generate various types of neurons for treatment of neurodegenerative diseases. Multiple inflammatory mediators and multiple immune/inflammatory cells such as mast cells are involved in the pathogenesis of neurodegenerative diseases. A better understanding of the brain and the peripheral immune and inflammatory intertwined pathways is imperative for the effective therapies of brain repair and regeneration.

## Conclusion

Although the mechanisms of neuroinflammation mediated neurodegeneration is not yet clearly understood, neuroinflammation is considered an important contributing factor for the neuronal death in the brain of age-related neurodegenerative disease. Peripheral immune response/activation and peripheral inflammation could generate several proinflammatory and neuro cytotoxic mediators. These peripheral inflammatory factors as well as peripheral inflammatory cells such as mast cells and T cells enter the brain. Further, these cells and inflammatory mediators could directly or through astrocyte, microglia, neurons and mast cell activation induce and sustain chronic neuroinflammation leading to neuronal death in the brain. Although suppression of neuroinflammation could reduce the severity of disease symptoms and neurodegeneration, the aging factor and immune disorder could continue to influence neurodegeneration. Therefore, novel approaches are required to better understand the communication pathways between the systemic and CNS immune system for the development of new and effective strategies to limit neurodegeneration and promote neuroregeneration in the brain.

## Author Contributions

DK and AZ wrote and critically edited the manuscript. RT, GPS, SZ, MEA, SPR, HZ, DS, PAN and SI edited the manuscript.

## Conflict of Interest Statement

The authors declare that the research was conducted in the absence of any commercial or financial relationships that could be construed as a potential conflict of interest.
